# Dual Infection of Hepatitis A Virus and Hepatitis E Virus— What Is Known?

**DOI:** 10.3390/v15020298

**Published:** 2023-01-20

**Authors:** Ibrahim M. Sayed

**Affiliations:** 1 Department of Biomedical and Nutritional Sciences, University of Massachusetts Lowell, Lowell, MA 01854, USA; ibrahim_ibrahim@uml.edu; 2 Department of Medical Microbiology and Immunology, Faculty of Medicine, Assiut University, Assiut 71515, Egypt

**Keywords:** viral hepatitis, HAV, HEV, dual infection, outcomes, treatment, prevention

## Abstract

Viral hepatitis is an infection of human hepatocytes resulting in liver damage. Dual infection of two hepatotropic viruses affects disease outcomes. The hepatitis A virus (HAV) and hepatitis E virus (HEV) are two enterically transmitted viruses; they are single-stranded RNA viruses and have common modes of transmission. They are transmitted mainly by the fecal-oral route and ingestion of contaminated food, though the HAV has no animal reservoirs. The HAV and HEV cause acute self-limiting disease; however, the HEV, but not HAV, can progress to chronic and extrahepatic infections. The HAV/HEV dual infection was reported among acute hepatitis patients present in developing countries. The impact of the HAV/HEV on the prognosis for acute hepatitis is not completely understood. Studies showed that the HAV/HEV dual infection increased abnormalities in the liver leading to fulminant hepatic failure (FHF) with a higher mortality rate compared to infection with a single virus. On the other hand, other reports showed that the clinical symptoms of the HAV/HEV dual infection were comparable to symptoms associated with the HAV or HEV monoinfection. This review highlights the modes of transmission, the prevalence of the HAV/HEV dual infection in various countries and among several study subjects, the possible outcomes of this dual infection, potential model systems for studying this dual infection, and methods of prevention of this dual infection and its associated complications.

## 1. Introduction

Hepatitis A virus (HAV) and hepatitis E virus (HEV) are enterically transmitted viruses that affect significant populations worldwide. HAV infection causes acute viral hepatitis that is associated with a mortality rate of 0.5% according to the WHO, 2016 [1]. The WHO reported that there are about twenty million HEV infections globally per year, 3.3 million clinically manifested patients, and there were 44,000 deaths in 2015 [2]. The WHO reported that 3.3% of mortality caused by viral hepatitis was attributed to HEV infection [2].

Both HAV and HEV have a genome of approximately 7.2–7.5 kb [3,4,5]. HAV is a positive sense single-stranded RNA virus; it belongs to the genus *Hepatovirus* and family *Picornaviridae* [6]. The HAV *genome* includes one open reading frame (ORF), untranslated region (UTR) at the 5′ terminus, and UTR and poly-A tail at the 3′ terminus [3]. The HAV genome encodes one large polyprotein that is processed into structural proteins and non-structural proteins [4]. HEV is a positive sense single-stranded RNA virus; it belongs to the family *Hepeviridae* which has two subfamilies *Orthohepevirinae* and *Parahepevirinae* [7]. The HEV genome encodes three ORFs and a cap at the 5′ terminus [8]. HEV ORF1 encodes non-structural proteins, and HEV ORF2 and HEV ORF3 encode structural protein and ion channel protein, respectively [5,8,9,10,11].

HAV has one serotype, and it has five genotypes (I-V). Genotypes I-III infect humans, and genotypes IV and V infect primates [12]. Human HAV isolates are subgenotyped into A and B (IA, IB, IIA, IIB, IIIA, and IIIB) [12]. HAV genotypes I and III are the most known genotype globally [13]. The HAV infects humans only, and there are no animal reservoirs for HAV. In contrast, humans and animals are the main reservoirs for HEV. The subfamily *Orthohepevirinae* includes four genera, two of them (*Paslahepevirus, Rocahepevirus*) including HEV isolates that infect humans [7]. The genus *Rocahepevirus* includes rat HEV isolates that cross species to humans [14]. The genus *Paslahepevirus* includes mammalian HEV isolates that are subdivided into eight genotypes [7]. Humans are susceptible to infections by HEV-1, HEV-2, HEV-3, HEV-4, and HEV-7 isolates, and there are no animal reservoirs for HEV-1 and HEV-2 isolates [15]. HEV-1 is subdivided into at least seven subtypes (1a–1g) and HEV-2 is subdivided into two subtypes (2a–2b) [16]. In contrast, there are animal reservoirs for HEV which infect humans, such as HEV-3, HEV-4, and HEV-7, and some genotypes have only been detected in animals, such as HEV-5, HEV-6, and HEV-8 [16]. Swine and wild boars are well-known sources of infection for HEV-3 and HEV-4 [17,18]. HEV-3 is subdivided into at least 13 subtypes (3a–3m) and HEV-4 is categorized into at least nine subtypes (4a–4i) [16]. Rabbits are also reservoirs for rabbit-derived HEV isolates which are close to HEV-3 [16,19]. Wild boars are sources of HEV-5 and HEV-6 [16]. Camels are reservoirs for HEV-7 and HEV-8 isolates; HEV-7 was found in dromedary camels, and HEV-8 was detected in Bactrian camels [20,21,22,23]. Humans are the common host for HAV and HEV [7,12].

HAV and HEV replicate in the liver and there are two forms of viruses produced during viral pathogenesis: naked or non-enveloped virions, which are present mainly in human stool, and quasi-enveloped viruses, which are present in human plasma [24,25,26]. A high viral titer was found in the stool, mediating virus transmission, and spreading the infection [24,25,26].

HAV and HEV share many similarities in genome structure, virus forms, pathogenesis, and mode of transmission (which will be discussed later in the review). In this review, we give an update on the literature reporting the dual infection of HAV/HEV, possible outcomes, and preventive measures to reduce the risk of this dual infection. Since in most literature, it is not clear if infection by both viruses occurred simultaneously or if one virus preceded the other, the term dual infection is used in the review to describe both situations.

## 2. Modes of Infection and Possible Source(s) of Dual Infection

**Figure 1:**
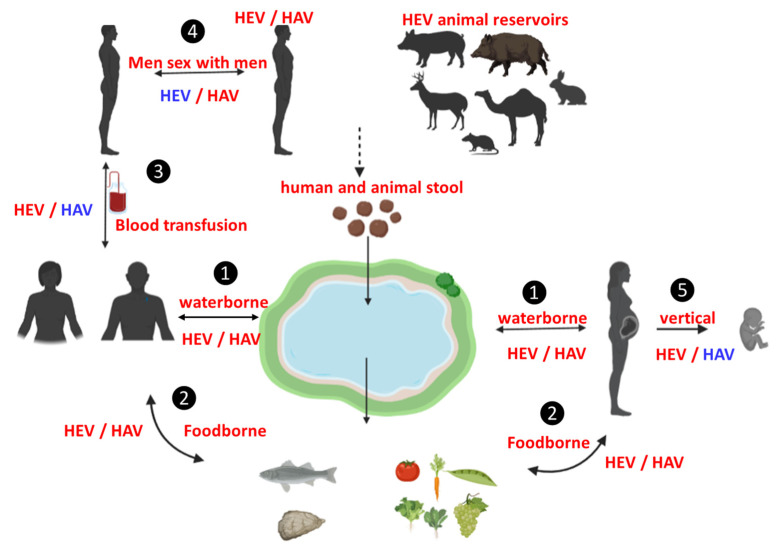
Possible routes of HAV/HEV dual infection. Humans are reservoirs for HAV and HEV, while pigs, wild boar, deer, and other animals are reservoirs for HEV only. (1) Waterborne dual infection has been confirmed for HAV/HEV via the fecal oral route. (2) Foodborne infection—ingestion of vegetables/fruits irrigated by contaminated water or seafood—has been confirmed for both viruses. (3) Blood transfusions, i.e., transmission via transfusion, have been confirmed for HEV (red) but are not a frequent source of HAV (blue). (4) Person-to person-contact such as MSM has been confirmed for HAV (red) but is not a frequent source of HEV (blue). (5) Vertical transmission, i.e., from mother to child, has been confirmed for HEV (red) but is not a frequent source for HAV (blue). For HEV/ HAV: Red means a confirmed source of infection; blue means not a frequent source, which is possible but not confirmed yet.

To predict the common modes of HAV/HEV dual infection, it is important to discuss the modes of transmission of each virus. Some modes of transmission are common to both viruses, while others are not (summarized in Figure 1). HAV and HEV infections are transmitted via contaminated water, which represents the fecal-oral route [15,27,28]. Foodborne infections are also caused by both viruses, but there are some differences. Foodborne HAV infections are common. Several products were associated with HAV-foodborne infections such as salads, sandwiches, multiple foods, fruits, and vegetables [29,30]. Recently HAV was detected in milk products and cheese [31,32]. Most foodborne-HAV infection is mediated through food handlers and restaurants and not animal reservoirs [29]. Foodborne HEV infections, caused by zoonotic genotypes, are mainly transmitted through ingestion of contaminated edible products from pigs, wild boars, deer, rabbits, and camels [19,33,34,35]. Also, ingestion of contaminated vegetables and fruits was associated with HEV infection. The water supply has been contaminated with either human or animal stool and has become a source of HEV transmission to these products during irrigation [30,36]. Importantly, HAV and HEV infections have been associated with the ingestion of seafood [37,38,39,40].

Person-to-person transmission through sexual contact or sharing drugs with others can greatly spread HAV infection [41,42]. The HAV outbreak has been associated with men who had sex with men (MSM) or who had oral-anal sexual contact [43]. However, HEV infection through person-to-person contact is not common; there are few such cases [44]. Teshale et al. reported that person-to-person transmission of the HEV-1 outbreak in Uganda was probably due to poor sanitation and hygiene [45]. Several studies reported that HEV is not transmitted among MSM or it has a low prevalence of transmission even during HAV outbreaks affecting MSM [46,47,48]. In these studies, there was no significant difference between anti-HEV IgG prevalence among MSM and control groups [46,47,48]. HEV RNA was not recorded in MSM, while two cases were anti-HEV IgM positive [46]. However, there is one study that reported the prevalence of HEV and HAV antibodies was significantly higher in MSM (14.6%) compared to the control group (1%), suggesting that MSM are at higher risk of being infected with the 2 pathogens and that HEV should be considered or investigated during HAV outbreak in the MSM population [49]. Anti-HEV IgM was recorded in 6.1% of MSA, but HEV RNA was not detected in any patients [49].

Few cases of HAV infection have been linked to blood transfusions, and this mode of transmission is rare [50,51]. On the other hand, HEV-3 and HEV-4 infections through blood transfusions were confirmed [52,53,54]. The HEV RNA was detected in blood products and associated with transfusion-transmitted HEV infections [52,53,55]. Dual infection by HAV/HEV was recorded in Israeli hemophiliacs. Higher anti-HAV seroprevalence was recorded in HEV-seropositive patients compared to HEV seronegative patients [56]. In contrast, there has been no difference between HEV seropositive and HEV seronegative patients in the level of bloodborne viruses markers including HBV, HCV, and HIV [56], suggesting that the fecal-oral route could be the mode of HAV/ HEV coinfection.

HAV infection can lead to complications in pregnant women such as premature labor, gestational complication, and acute liver failure [57,58]. However, the transmission of HAV infection from mother to fetus during pregnancy is controversial. Few cases of intrauterine HAV transmission from a pregnant woman have been documented with the fetus developing complications such as ascites, polyhydramnios, and meconium peritonitis [57,59]. In contrast, other studies showed that HAV infection during pregnancy did not impact the fetus, and the fetus tested negative for HAV markers [57,60]. Transmission of HEV from mother to child is confirmed, and the outcomes of HEV infection depend on virus genotype [61,62]. HEV-1, HEV-2, and HEV-4 have adverse feto-maternal outcomes and maternal fatality [62,63,64,65]. HEV-3 causes a mild course of infection with either low complications or none for both mother and child [66].

Collectively, it seems that the fecal-oral route or waterborne infection is the most common method of HAV/HEV coinfection. Although there is no report on dual infection through foodborne infection, both viruses have been detected solely in some common products such as fruits, vegetables, and seafood. Person-to-person contact, especially through MSM and blood transfusions are other potential ways of transmitting HAV/HEV coinfection, but further studies are needed to confirm these modes.

## 3. Incidence of Dual Infection HAV/HEV

The dual infection by HAV and HEV has been confirmed in many developing countries, and to a small extent in developed countries (Table 1). In developing countries, HAV/ HEV coinfections were reported in India, Mexico, Kenya, Bangladesh, and Egypt (Table 1). HAV/HEV dual infections were associated with several outbreaks in Cuba; the number of HAV/HEV outbreaks was higher than the number of outbreaks caused by HEV alone [67]. In addition, a water-borne outbreak was reported in Hyderabad, where HEV infection caused 78.5% of infections, and HAV/HEV mixed infection was detected in 5.3% of the infected population [68]. In India, several studies assessed the rate of HAV/HEV coinfection, and the results were variable depending on the studied groups, geographic distribution, age, etc. Surveillance was conducted by large laboratories on 24,000 patients during 2014–2017 to assess the prevalence of HAV and HEV. The infection rate was 16.1%, 12.6%, and 1.3% for HEV, HAV, and dual infection (HAV/HEV), respectively [69]. Another study was retrospectively conducted on acute hepatitis patients, pregnant women, and non-pregnant women. HAV/HEV dual infection was detected in 2.07% of acute hepatitis patients, 2.6% among males and 1.7% among females [70]. Additionally, dual infection was detected in 2.5% of non-pregnant women and 1.2% among pregnant women [70]. Liver function values were more abnormal in dual infection cases compared to monoinfection with either HEV or HAV [70]. In some regions in India, HEV/HBV dual infection was the most common haptotropic dual infection among all viral hepatitis cases [71], while in other regions HAV/HEV dual infection was the most prevalent dual infection [72]. Moreover, some studies reported that HAV/HEV dual infection was predominant in children [71], while others showed that adults were more affected by HAV/HEV dual infection [73]. The incidence of HEV/HAV dual infection reported in several studies in India is summarized in Table 1. HEV infection is the most common viral hepatitis detected in India, and the infection is mainly associated with waterborne transmission [74]. Therefore, the proposed mode of HAV/HEV dual infection there is mediated via the fecal-oral route. In Bangladesh, diagnosis of acute hepatitis patients (n = 1925) during the period 2014–2017 revealed that HEV was the most common cause. The mortality rate among acute HEV infections was 5% and increased to 12% among pregnant women. The highest risk of mortality among HEV-infected patients was through dual infection with HBV or HAV [75]. In Egypt, most of the dual infected HAV/HEV cases were children [76,77,78]. HAV/HEV dual infection was confirmed in 70 (26%) out of 268 children who developed acute viral hepatitis [76]. Several studies revealed that HAV circulating in drinking water treatment facilities and sewage in Egypt, where the isolated viruses belonged to the HAV genotype IB, was the same strain isolated from clinical patients [79,80]. HEV RNA was also detected in water treatment plants in Egypt [81]. Therefore, it seems that water is the main source of the HEV/HAV dual infection in Egypt either directly or through using it for treatment and irrigation of plants, vegetables, or fruits; the dual infection may be transmitted via ingestion of these contaminated products. In Venezuela, HAV/HEV dual infection (31%) was slightly higher than HEV monoinfection (29%) among acute hepatitis patients of various ages. Phylogenetic analysis performed on some HEV isolates revealed that HEV genotype 3 was the isolated strain from one of the coinfected cases, while HAV isolates did not characterize the study [82]. The coinfected patient had abnormal liver values. In Mexico, HAV is endemic and HAV/HEV dual infection was reported. The circulating HEV strains in the dual infection were found; they belonged to the HEV genotype 1 [83]. In China, among 46 acute hepatitis patients, five patients (10.87%) were HAV/HEV dual infected. The coinfected patients had higher liver transaminases, bilirubin, and jaundice compared to HEV or HAV-monoinfected patients [84]. The isolated HAV was genotype 1A, but HEV had not been characterized in this dual infection [84]. In addition, HAV, HEV, and HAV/HEV dual infections were assessed in animals present there. HEV RNA was detected only in rabbits and pigs, and HAV was detected only in ferrets, while dual infection HEV/HAV was not detected [84]. In Italy, HEV/HAV dual infection (14%) was recorded in MSM [49]. In Israel, high anti-HAV antibodies, but not HBV or HCV, were recorded in HEV seropositive hemophiliac patients, suggesting a possible HAV/HEV dual infection among those patients [56].

## 4. Outcomes of Dual HAV/HEV Infection

Both HAV and HEV are hepatotropic and they replicate mainly in human hepatocytes. Therefore, dual infection or superinfection could impact the viral pathogenesis in the liver and disease outcomes.

### 4.1. Clinical Symptoms of HEV or HAV Monoinfection

HEV or HAV infections are acute self-limiting diseases with comparable symptoms, including gastrointestinal, hepatitis, and/or fever. However, there are some differences in the clinical outcomes between HEV and HAV monoinfection. HAV infection does not develop chronicity, while HEV infection, especially genotype 3 and genotype 4, develops into a chronic infection, especially in immunodeficient patients such as AIDS patients, organ recipients, and patients with hematological diseases [102,103]. Besides, HEV-infected patients could develop extrahepatic disorders such as renal, neurological, and blood disorders [104,105,106]. Extrahepatic disorders are rare in HAV-infected patients, which include renal, arthritis, cutaneous vasculitis, and neurological diseases [107,108,109,110]. HEV infection could result in fulminant hepatic failure (FHF) [100,111], while FHF is rare in HAV monoinfection [112,113].

### 4.2. Impact of Dual Infection (HAV/HEV)

The impact of dual HAV/HEV infection on disease outcome compared to monoinfection is controversial. Some studies showed that dual infection does not affect the disease pathogenesis, while others reported that dual infection results in severe disease outcomes. Kaur and colleagues reported that dual infection of HEV with other hepatotropic viruses such as HAV, HBV, and HCV was common in India, and there were no differences in symptoms, clinical profile, and disease prognosis between patients infected with a single virus or dual infected patients [114]. Similarly, Kumar et al. assessed hepatotropic viruses (HAV, HBV, HCV, and HEV) and evaluated the impact of dual infection among children diagnosed with acute viral hepatitis (n = 122) and patients who developed FHF (n = 27). There were no differences between HAV/HEV dual infected children (n = 24) and children infected with a single virus in all clinical parameters analyzed, such as jaundice incidence and duration, disease recurrence, ascites, mortality rate, etc. [115]. Similarly, there was no observable difference between monoinfection and HAV/HEV dual infection in the FHF group [115]. The authors hypothesized that dual infection with two or more hepatotropic viruses did not lead to a severe outcome, and HEV infection/seropositivity was linked to 88% of dual infection cases in acute viral hepatitis patients [115]. Similarly, Sarguna and colleagues evaluated the causative agents and outcomes associated with the waterborne outbreak that occurred in Hyderabad [68]. Out of 546 patients, 429 patients were infected with HEV, 53 patients were infected with HAV, and 29 patients were dual infected with HEV/HAV. There was no difference in clinical symptoms and liver function tests between coinfected patients and monoinfected patients. Most patients recovered without complications [68].

However, other studies reported that HEV/HAV dual infection can lead to severe outcomes. Arora et al. assessed the causative agent of acute liver failure in children, and importantly, the authors reported that dual infection by the HAV/HEV (20.45%) was the main cause of FHF in children, of which three children died. The rate of acute liver failure (ALF) caused by dual infection HAV/HEV was higher than ALF caused by a single agent (9% for HAV and 13.6% for HEV) [85]. Several cases of HAV/HEV dual infection developed acalculous cholecystitis, hepatic encephalopathy, and the worst outcomes [116,117,118]. Other studies supported the finding that the HEV/HAV dual infection can lead to a severe course of the disease as shown by abnormalities in liver function tests or liver abnormalities [70,84,119]. Likewise, Paul and colleagues reported that the fatality rate associated with HEV infection was increased in pregnant women, as was the presence of dual infected hepatotropic viruses, especially HBV or HAV [75].

The reasons for the discrepancies among the previous reports about the impact of HAV/HEV on the disease outcomes are not clear, but they could be attributed to different study subjects, geographical locations, risk factors, or viral genotypes, etc.

### 4.3. HAV/HEV Dual Infection with Another Hepatotropic Virus

HAV/HEV dual infected patients could also be infected with another hepatotropic pathogen such as HCV and HBV. These cases are rare, and few cases were described in the literature. Butt and colleagues described a 12-year boy’s case of acute viral hepatitis, where the boy was coinfected with three hepatotropic viruses, HAV, HBV, and HEV [120]. The case had elevated liver transaminases, but there were no severe complications associated with the case, suggesting that the three hepatotropic viruses did not affect each other [120]. Poddar and colleagues reported another acute hepatitis case in which the patient was coinfected with HAV, HEV, and HCV [87]. The authors did not mention the outcomes of this case. Since these cases are few, we could not draw conclusions on the impact of the pathogenesis of three hepatotropic viruses on the liver. Importantly, the HEV superinfection on chronically HBV-infected patients could lead to a higher mortality rate; the coinfected patients did not respond to anti-HBV therapies and progressed to liver failure [121]. Future studies should assess the outcomes of infection by three hepatotropic viruses.

## 5. Preventive Measures for HAV and HEV Dual Infection

There is no specific therapy or treatment for acute HAV or HEV infections; only symptomatic and supportive therapies are required [5,122]. The HAV post-exposure prophylaxis includes immunoglobulin and/or a vaccine [123]. Both measures can be given to immunocompromised patients and patients with chronic liver disease [123]. The HAV vaccine is recommended for patients aged 1–40 years, while immunoglobulin is recommended in children less than 12 months or in cases of allergy to the vaccine [123]. Non-specific therapies such as ribavirin and interferon are recommended for chronic HEV infections [124]. However, to our knowledge, most of the reported HAV/ HEV dual infections were acute, not chronic infections (Table 1). Therefore, there is no specific therapy for dual HAV/HEV infections. Preventive measures seem to be the best strategy to reduce the risk of dual HAV/HEV infections and their complications. Since most of the reported dual HAV/HEV infections were linked to the fecal-oral route or waterborne infections, improving hygiene and sanitation practices could reduce the risk of dual infections. Also, frequent washing of hands, vegetables, and fruits could reduce the spread of viruses. The HAV has a vaccine that is given to children, which induces long-lasting immunity. Since most HAV/HEV dual infection was reported in children, the HAV vaccine could reduce the infection rate and virus transmission. However, a recent study showed that some HAV antigenic variants can escape vaccine-mediated immune responses [125]. Hecolin is an approved HEV vaccine in China. This vaccine showed full protection against HEV-4 and partial protection against HEV-C1 infection [126,127]. By conducting a clinical study on four volunteers, Wen and colleagues showed that Hecolin can induce antibodies which could react with several HEV genotypes [128]. Preventive measures during MSM or oral-anal intercourse should be considered to reduce or prevent the infection.

## 6. Challenges in HAV/HEV Dual Infection Research and Future Perspectives

As mentioned in the previous section, the outcomes of HAV/HEV dual infection are questionable. Limited sequences of HAV and HEV isolates were characterized the dual infections. It is not known if different HAV and HEV genotypes and subtypes can affect the outcome of liver diseases. Huh7-A-I cells can be used for the growth of the wild-type HAV virus [129]. The HAV can adapt to the cell culture after several sub-passages. Highly adapted HAV isolates can replicate efficiently in the cell culture, causing a cytopathic effect and apoptosis in the infected cells [130,131]. HEV genotypes 1 and 2 are the main genotypes associated with waterborne infections [15]. However, the growth of HEV genotype 1 in the hepatoma cell line is limited [132,133]. Therefore, it seems that there is no general culture system for studying HAV/HEV dual infections. A recent study showed that dual infection of HCV with HEV leads to viral interference [134]. Using Huh7.5 cells and the HCV/HEV co-transfection model, HEV replication was reduced, while HCV replication was not altered [134]. HCV infection inhibits HEV replication via HCV protease NS3/4A, which probably cleaves HEV ORF1 to a less active form [134]. On the other hand, the HAV/HCV dual infection with Huh7.5 showed that there were no direct interactions between the two viruses and limited competition [135]. Future studies should study the impact of HAV/HEV dual infection in vitro.

Studies showed that immunodeficient humanized mice (uPA-SCID or FRG) are suitable models for studying HEV infections, especially HEV genotypes 1 and 3 [136,137,138,139,140,141]. HEV replicates in the human hepatocytes occupied in the murine liver and the produced viral particles are excreted in the stool and blood of the infected mice [136,137,138,139,140,141,142]. The replication of HEV in these models is non-cytopathic, i.e., no damage occurs to the human hepatocytes [137]. HEV particles released in the mouse stool were nonenveloped, while HEV particles released in the blood were enveloped [137]. Moreover, uPA-SCID humanized mice were used to study the innate liver transcriptome against the HEV genotype 1 infections [137]. Interestingly, Hirai-Yuki and colleagues showed that uPA-SCID humanized mice can be used for studying the propagation and pathogenesis of HAV [143]. Similar to HEV infection in these mice, HAV replicates specifically in human hepatocytes and the propagated viruses were released in stool as naked viruses and in the blood as quasi-enveloped particles [143]. HAV infection is not cytopathic in these mice and infection induces host interferon responses [143]. Researchers can utilize the humanized mouse model for studying the impact of dual HAV/HEV infections. Humanized mice can be challenged with HAV (monoinfection), HEV (monoinfection), or both viruses (HAV and HEV) via intraperitoneal or intravenous injection. The viral load can be assessed in the mouse plasma and stool, and the viral proteins can be detected in the humanized mouse liver. The impact of dual infection HEV/HAV on the transcriptome changes of hepatocytes and its effect on liver pathogenesis can be compared with monoinfection in this model.

## 7. Conclusions

The HAV/HEV dual infection is common in developing countries; it is mainly transmitted via the fecal-oral route and may be associated with outbreaks. The impact of this dual infection is questionable, and more research should be conducted in the future to assess the impact of dual infection. Improving practices in hygiene could reduce this dual infection and possible complications.

## Figures and Tables

**Table 1:** The rate of HAV/HEV dual infection among different groups in different countries.

Country		Study Groups and Number	Method of Analysis	Results and Dual Infection Rate	Ref
India	New Delhi	Acute liver failure, children, (n = 44)	Anti-HAV IgM Anti-HEV IgM HEV RNA Immunoblot assay for anti-HEV antibodies.	HEV/HAV dual infection was the main cause of fulminant hepatic failure (FHF) (20.45%), followed by HEV (13.6%). HAV coinfection was only associated with 9% of infection.	[85]
	Dibrugarh, Assam	Acute hepatitis, adults (14–55 age), (n = 591)	Anti-HAV IgM Anti-HEV IgM	195 cases (33%) were infected with HAV, from whom 26 cases (4%) developed acute liver failure and 17 cases (3%) died. 121 cases (20%) were infected with HEV, from whom 19 cases (3%) developed acute liver failure and 11 cases (2%) died. 9 cases (2%) were infected with combined infection, from whom 1 patient (0.1%) developed acute liver failure and died. HAV is the most common cause of viral hepatitis	[86]
	Chandigarh	Acute hepatitis, children (<14 years of age), (n = 172)	Anti-HAV IgM Anti-HEV IgM	HAV caused 64.5% of infections. HEV caused 16.3% of infections HEV/HAV dual infection caused 7% of infection. HEV/HAV/HCV mixed infection was recorded in 1 patient	[87]
	south India	Acute hepatitis, children (n = 127)	Anti-HAV IgM Anti-HEV IgM	38.6% HAV monoinfection. 15.7% HEV monoinfection Dual infection of HEV/HAV was developed in 17 patients (13.4%).	[88]
	South India, Vellore	Acute hepatitis, children, and adults (n = 404)	Anti-HAV IgM Anti-HEV IgM	HAV infection was detected in 13.3% and HEV infection was detected in 17.3%. Dual HEV/HAV infection was detected in 3 patients (0.8%), all of them were male.	[89]
	North India (Lucknow, Uttar Pradesh)	Acute viral hepatitis, children, and adults (n= 267)	Anti-HAV IgM Anti-HEV IgM	26.96 % of infection due to HAV. 17.97% of infection due to HEV. HEV/HAV dual infection was detected in 15 cases (7.3%) of acute hepatitis patients (both children and adults). HEV/HAV was the most common dual infection among hepatotropic pathogens	[72]
	West Bengal	Acute hepatitis, children, and adults (n = 285)	Anti-HAV IgM Anti-HEV IgM, IgG antibodies HEV RNA	17.2 % of infection due to HAV. 34.4% of infection due to HEV. Dual HEV/HAV infection was detected in 2.1% of the patients, which was lower than HEV/HBV dual infection (3.8%), but higher than HEV/HCV (0.35%)	[90]
	Jabalpur, Madhya Pradesh	Acute hepatitis, children, adults (n = 555)	Anti-HAV IgM Anti-HEV IgM	5.1% of infection due to HAV. 13.7% of infection due to HEV. HEV/HAV was reported in 8 cases, 6 cases in children at or below 14 years and 2 cases in age 15–45 years.	[71]
	Hyderabad	Acute hepatitis, children, adults (n= 546) during water outbreak	Anti-HAV IgM Anti-HEV IgM	9.7% of outbreak due to HAV. 78.5% of outbreak due to HEV. 5.3% of the outbreak was due to mixed HEV/HAV infection.	[68]
	Mangalore	Acute hepatitis, children, adults (n =958)	Anti-HAV IgM Anti-HEV IgM	127 cases were monoinfected with HAV. 58 cases were monoinfected with HEV. HEV/HAV dual infection was detected in 33 cases (11.5%)	[91]
	Northwest Districts of Punjab	Acute hepatitis, children, adults (n = 95). It is composed of 9 outbreaks	Anti-HAV IgM Anti-HEV IgM	Two cases (2.1%) were infected with HAV. 65 (68.42%) cases were infected with HEV. The patients in the age of 21–30 years old HEV/HAV dual infection was detected in 6 cases (6.31%). The coinfected patients from all ages	[74]
	Uttarakhand	Acute hepatitis, children, adults (n = 617).	Anti-HAV IgM Anti-HEV IgM	HAV and HEV were detected in 14.7% (91/617) and 28.04% (173/617), respectively. Dual infection of HEV/HAV was recorded in 32 out of 617 cases (5.2%).	[92]
	Northern India.	Acute hepatitis, children, adults, and pregnant (n = 5319)	Anti-HAV IgM Anti-HEV IgM	HAV and HEV was found in 16.9% and 14.9% of infections, respectively. Dual infection of HEV/HAV was detected in 1.6% of cases. The frequency of dual infection were adults > pregnant women > children	[73].
	Western India, Pune, Maharashtra	Acute hepatitis, children, adults. (n = 1807)	Anti-HAV IgM Anti-HEV IgM	HAV and HEV was found in 6.7% and 8.5% of infections, respectively. HEV/HAV dual infection was 0.6% and associated with severe complications	[93]
Bangladesh		Hospitalized acute jaundice patients, adults and pregnant (age > 14 years), (n = 1925)	Anti-HAV IgM Anti-HEV IgM Anti-HEV IgG HEV RNA	HEV monoinfection was the main cause (34%) and a mortality of 5% among jaundice patients and 12% among pregnant women. Dual infection of HAV/HEV or HBV/HEV were risk factor of mortality.	[75]
		Fulminant hepatic failure, adults (n = 67)	Anti-HAV IgM Anti-HEV IgM	43 patients (64%) were infected with HEV only. 4 patients (6%) were HEV/HBV dual infected, and 3 patients (4.5%) were HEV/HAV dual infected.	[94]
		Acute jaundice syndrome (AJS) among the Rohingya refugees in Cox’s Bazar, an outbreak affecting all ages (n = 275)	Anti-HAV IgM Anti-HEV IgM	HEV/HAV was recorded in one case (0.4%), which was lower than HAV/HBV dual infection (4%) and HAV/HCV dual infection (0.7%).	[95]
China		Acute hepatitis patients with either HAV or HEV infection (n = 46). Fecal samples from 11 animal species (n = 675)	Anti-HAV IgM HAV RNA Anti-HEV IgM HEV RNA	HEV/HAV dual infection was found in five patients (10.9%). The isolated HAV was genotype 1A, HEV was not characterized in the dual infection. No dual infection in animals. HEV was detected in rabbits and pigs. HAV was detected in 1% of ferret.	[84]
Korea		Acute hepatitis, adults, (n = 55)	Anti-HAV IgM Anti-HEV IgM	HAV and HEV was found in 56.4% and 9.1% of infections, respectively. HEV/HAV dual infection was detected.	[96]
Mexico		Acute hepatitis, children	Anti-HAV IgM HAV RNA Anti-HEV IgM HEV RNA	HAV was 100% and no HEV (0%) monoinfection. HAV/HEV dual infection was 58% in the West and 10% in the South	[97].
Venezuela		Acute hepatitis, children, and adults (1–55 years), (n = 39)	Anti-HAV IgM Anti-HEV IgM HEV RNA	HEV/HAV dual infection (31%) was slightly higher than HEV monoinfection (29%). HEV genotype 3 in dual infection with HAV. HAV was not characterized	[82]
Italy		Men who have sex with Men (MSM), adults (20–85 years), (n = 636)	Anti-HAV IgM Anti-HEV IgM Anti HEV IgG HEV RNA	The seroprevalence of HEV/HAV was significantly higher (14.6%) in MSM compared to control group (1%),	[49]
Mitrovica		Acute hepatitis, children, epidemics, aftermath of the war in Kosovo (1999) (n = 104)	Anti-HAV IgM Anti-HEV IgM	HAV caused 88% of infection. HEV caused 8/104 (7.7%) of infection. HEV/HAV dual infection was detected in 2 out of 8 cases of HEV, and they were symptomatic and sick. Epidemic in school due to drinking of well water	[98]
Egypt		Acute hepatitis, children, (n = 268)	Anti-HAV IgM Anti-HEV IgM	HEV/HAV dual infection was confirmed in 70 (26%).	[76]
		Acute hepatitis, children, (n = 180)	Anti-HAV IgM Anti-HEV IgM Anti-HEV IgG HEV RNA	HEV markers were positive in 34% of HAV infected children. There is association between HEV and HAV infections.	[77]
		Acute hepatitis, children, adults, aged (1–73 years), (n = 202)	Anti-HAV IgM Anti-HAV IgG HAV RNA Anti-HEV IgM Anti-HEV IgG HEV RNA	10.4% of cases were anti-HAV IgM positive and 99.5% were anti-HAV IgG positive. Anti-HEV IgM and anti-HEV IgG were recorded in 24.2% and 44.5% of patients, respectively. Dual infection HEV/HAV was recorded in three patients (1.5%)	[99]
		Acute hepatitis, children, (n = 73)	Anti-HAV IgM Anti-HEV IgM	HAV and HEV were found in 41% and 12%, respectively. Dual infection of HAV/HEV was recorded in two cases (2.74%)	[78]
		Acute hepatitis patients, aged (14–64 years), (n= 18)	Anti-HAV IgM Anti-HEV IgM	Dual infection of HAV/HEV was found in two cases (11.1%)	[100]
Kenya		Acute hepatitis patients (n = 100)	Anti-HAV IgM Anti-HEV IgM	one patient (1%) was dual infected with HEV/HAV	[101]

## Data Availability

Not applicable.
